# Peer Review of “Levels and Predictors of Knowledge, Attitudes, and Practices Regarding Contraception Among Female TV Studies Undergraduates in Nigeria: Cross-Sectional Study”

**DOI:** 10.2196/72951

**Published:** 2025-05-08

**Authors:** Bilkisu Nwankwo

**Affiliations:** 1 Kaduna State University, Tafawa Balewa Way, Kaduna State, Nigeria, 234 813 894 1459

**Keywords:** knowledge, attitudes, practice, contraception, regression, cross-sectional, females, students, Nigeria

*This is the peer-review report for “Levels and Predictors of Knowledge, Attitudes, and Practices Regarding Contraception Among Female TV Studies Undergraduates in Nigeria: Cross-Sectional Study.”*

## Round 1 Review

### Specific Comments

#### Major Comments

1. The sampling technique used in this paper [1] should be more detailed than it is. Respondents were said to have been selected by balloting from the 6 levels. Was it equal allocation per level, or was it proportionate allocation considering that it is not likely that there were the same number of students in each level?

2. State the age ranges of a teenager and that of a young adult in your methodology that informed the categorization in the Results.

3. Living with a spouse and not living with a spouse was considered for marital status in your study as opposed to being single, married, etc. Clarify why this is so.

4. The public health implications of some of the findings were omitted in the Discussion. This should be included. Its importance cannot be overemphasized.

#### Minor Comments

5. Abstract: The last sentence in the Methods is hanging. Kindly complete it.

6. Grammatical issues: Tenses: Future and present tenses were used where past tense should have been used in the methodology (lines 12 and 28). Present tense was used in multiple places in the Discussion where past tense should have been used.

7. Reference list: In the Vancouver referencing style, the month of publication should not appear as it did in some references like 7, 11, and 12. The date accessed/cited was written in some and not in others like 9, 10, 13, and 16. Really old references like reference 24, which is 14 years old, should be replaced by more current ones.
